# Networking between community health programs: a case study outlining the effectiveness, barriers and enablers

**DOI:** 10.1186/1472-6963-12-206

**Published:** 2012-07-19

**Authors:** Nathan J Grills, Priscilla Robinson, Maneesh Phillip

**Affiliations:** 1 Nossal Institute for Global Health University of Melbourne, Level 4, Alan Gilbert Building, 161 Barry St, Carlton, Victoria, 3010, Australia; 2 Department of Epidemiology and Preventive Medicine, Monash University, Melbourne, VIC, Australia; 3 School of Public Health, La Trobe University La Trobe University City Campus, 215 Franklin Street, Melbourne, Victoria, 3000, Australia; 4 Public Health Masters Student London School of Tropical Medicine and Hygiene, Keppel Street, London, WC1E 7HT, UK

## Abstract

**Background:**

In India, since the 1990s, there has been a burgeoning of NGOs involved in providing primary health care. This has resulted in a complex NGO-Government interface which is difficult for lone NGOs to navigate. The Uttarakhand Cluster, India, links such small community health programs together to build NGO capacity, increase visibility and better link to the government schemes and the formal healthcare system. This research, undertaken between 1998 and 2011, aims to examine barriers and facilitators to such linking, or clustering, and the effectiveness of this clustering approach.

**Methods:**

Interviews, indicator surveys and participant observation were used to document the process and explore the enablers, the barriers and the effectiveness of networks improving community health.

**Results:**

The analysis revealed that when activating, framing, mobilising and synthesizing the Uttarakhand Cluster, key brokers and network players were important in bridging between organisations. The ties (or relationships) that held the cluster together included homophily around common faith, common friendships and geographical location and common mission. Self interest whereby members sought funds, visibility, credibility, increased capacity and access to trainings was also a commonly identified motivating factor for networking. Barriers to network synthesizing included lack of funding, poor communication, limited time and lack of human resources. Risk aversion and mistrust remained significant barriers to overcome for such a network.

**Conclusions:**

In conclusion, specific enabling factors allowed the clustering approach to be effective at increasing access to resources, creating collaborative opportunities and increasing visibility, credibility and confidence of the cluster members. These findings add to knowledge regarding social network formation and collaboration, and such knowledge will assist in the conceptualisation, formation and success of potential health networks in India and other developing world countries.

## Background

According to Community Health expert Dr Ted Lankester, the idea of sharing amongst community health programs:

"“…struck me as blindingly obvious that if groups could work together to learn from each other, stop competing, start cooperating, combine resources, they would have greater credibility, advocacy power, ability to pull in donors and strength to demonstrate their combined value to government: that it was inevitably a sensible idea to help facilitate clusters. The frequent comments at the WHO consultation about the need for NGOs to come together so that governments and donors could relate to them, was a confirmation of this.”"

If it is blindingly obvious, then why do networks not just naturally form? What are the barriers and what factors assist network formation? When a network forms what are the benefits?

There is evidence on the effectiveness of networks in developed countries. Various social capital theorists use network analysis to describe the benefit derived from networks [1-3]. Alexander describes how networking can be effective at outcomes-based advocacy, vision-focus balance, systems orientation, infrastructure development, and community linkages [4]. Other studies show that information is effectively disseminated and resources generated [5-9]. However, these studies are not based in developing world health settings, and our literature review found little research relating to the health field in developing countries [10]).

Factors described as facilitating effective health networking include effective leadership [11], bridging nodes between organisations ([12], p.30), and brokerage where behaviours are closely managed, people from diverse backgrounds are synthesized together, effective communication is engendered and operating rules developed ([8], p.15). Homophily, or the tendency of individuals to associate and bond with similar nodes, has been shown to be important in facilitating networks [5,12-15]. McPherson, Smith-Lovin and Cook (2001) cite over one hundred studies that have observed homophily in some form or another: including age, gender, class and organisational role [14].

Another body of literature shows that individuals join networks for the actual or perceived benefits resulting from particular interrelations [7,8], ([12], p.30). The literature on networks is replete with theories about their potential benefits and effectiveness. For example, Weiss’s (1987) field research, described in Krueathep [9] explored the motivations for public administrators to collaborate, and she discovered four key areas of self interest: access to increased resources, the reduction of uncertainty, assistance in obeying legal mandates and political advantages.

The reasons that programs join networks may differ from their reasons for maintaining or increasing involvement in the network [5,12]. Furthermore, at each stage in a network’s life different factors will play a more or less important role in facilitating or retarding the network. Factors found to often reinforce the network include respect, mutual understanding and shared purpose such as tackling a wicked problem [12,16,17].

The process and stages of network formation and growth has been outlined by Agronoff and McGruire [16] in “Getting results through collaboration” by Mandell. They describe a fourfold typology representing stages, or sequences of network management [16]: network activation, framing, mobilisation and synthesizing.

This research explores the factors that facilitate and impede network activation, framing, mobilisation and synthesis. The article utilises the Uttarakhand Cluster network of programs to explore the facilitators and barriers to network formation and function. A thematic analysis is undertaken using a triangulation of indicator surveys, focus group discussions and participant observation. These findings will inform recommendations for future networks between Community Health NGOs.

The fieldwork, based largely in India, draws on international expertise from Melbourne University, the Community Health Global Network and the WHO Partnerships Division of the Director General's Office of the WHO. These international links will inform the research and assist in dissemination and transfer of relevant findings.

## Methods

India was selected as a case study as it represents a fertile context in which to explore community health networks given the diversity and density of community health NGOs and the dependency of the health care system on such providers [18,19]. Although the government system has grown rapidly under the National Rural Health Mission (see Table 1), 78% of outpatient services are still provided by non-government sources: often disparate and small NGOs [20].

**Table 1:** The different stages in network development

**Stage**	**Definition**	**Role of the Network manager**
*Activation*	Identifying participants and network stakeholders, directing their skills, knowledge, and resources [16,21]	Arranging, stabilising and nurturing the network structure [16]
*Framing*	Establishing the operating rules of the network, [16]	Influencing its prevailing values and norms and perceptions of the network participants
*Mobilising*	Generating and building commitment for the network and its purposes. To achieve this, nodes must be able to understand strategic whole and work towards common objectives based on the whole ([22], p.33)	Induce individuals to commit to a joint undertaking or specific network activities
*Sythesizing*	A blending of “various participants- each with their conflicting or different perceptions or dissimilar values” in order to work towards the network’s purpose ([13], p.15)	Enhance conditions for favourable, productive interaction amongst network participants

In Uttarakhand alone the Office of the Registrar estimated that there were 41,826 NGOs [23]. This is the ideal context in which to explore the benefit of clustering amongst non-state community health programs.

The Uttarakhand Cluster is a sub branch of the Community Health Global Network (http://www.chgn.org) which is a UK based charity which works by facilitating the formation of networks of community health programs, such at the Uttarakhand Cluster, for coordination, mutual support and the sharing of resources amongst CHGN members in relational, geographically focused groups.

In late October 2008 CHGN facilitated the inauguration of the Uttarakhand Cluster. Seventeen NGOs came together for a workshop labelled Linking-2-Learn, where they explored how they could creatively work together to build capacity. They co-signed a declaration of cooperation and decided on an action plan.

Ethics approval was obtained through La Trobe University to undertake interviews, focus group discussions and participant observation.

**Figure 1:**
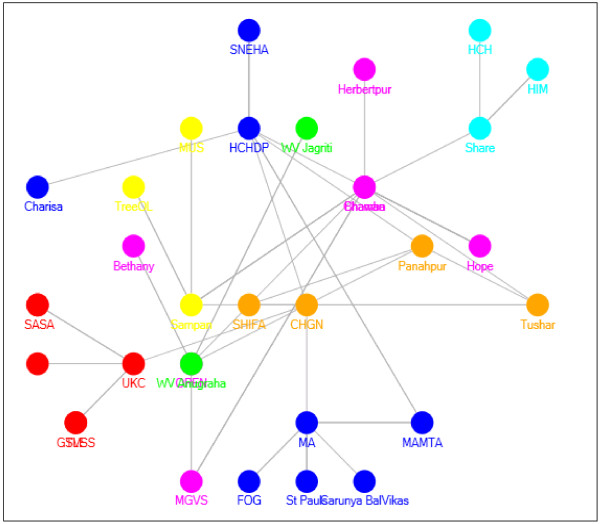
Sociogram depicting collaboration amongst programs in the cluster.

All member programs were included in the research to avoid selection bias. The baseline data collection involved mapping pre-existing linkages by undertaking an indicator survey to record the professional linkages between the programs. A linkage or tie with another NGO was counted either as a formal meeting, attending a joint training or being involved in a joint funding application. This was then plotted (see Figure 1).

Informal linkages between organisations were then explored qualitatively using a triangulation of methods which were analysed using thematic analysis. The methods used to explore these various relationships were Focus Group Discussions (FGDs), indicator surveys and participant observation. Participant observation (PO) consisted of field notes taken by the cluster facilitator on cluster activities including board meetings and general cluster meetings; correspondence between cluster members and the facilitators; and finally other important cluster documents.

FGDs were undertaken each year to coincide with the annual network meeting. In order to assist open discussion and avoid power differentials, two separate FGDs were undertaken. The first included the entire eight members of the Uttarakhand Cluster board and was conducted in English. The second FGD was conducted in Hindi with eleven program leaders in year one, and thirteen in year two, by recruiting the NGO leader, or consultant, from each cluster member. There was significant homogeneity in terms of demographics and status between FGD participants.

An outside facilitator was employed in order to limit bias. Each FGD was recorded and transcribed by an outside independent researcher. The results were combined with interview and participant observation data and then thematically analysed. This paper mainly utilises the first year of the data but the analysis will be completed at the conclusion of four years.

All the data was analysed and organised under the categories of:

1) The effectiveness of networking

2) The barriers to networking

3) The facilitators of networking

Thematic analysis was undertaken utilising an a priori framework developed from the literature to categorise the different factors under each heading.

## Results

The components that facilitated activation, framing, mobilising and synthesis are recorded under a framework that was iteratively adapted into themes which accounted for the findings. The themes are grouped under nodal factors, ties and homophily and ties and self interest”

### Nodal’ factors

A node is a network player and each person or institution involved in the network is considered a node.

#### The brokers

The data revealed that network brokers were crucial in network formation. Prior to the inaugural meeting the broker had undertaken months of targeted planning which was seen to be key in helping programs which, prior to the brokerage, co-existed for many years without substantively engaging with each other:

…we have all known one another since fifteen to twenty years, but we are working together for the very first time. Before this we have never heard one another’s problems. FGD-A1

The two external brokers, or “vision holders”, were credited with having animated the cluster by actively recruiting programs:

I think the most important factor in the initial success of the first meeting was meeting with most of the cluster participants individually prior to the meeting. This was a chance to sell the vision, to ask them to be involved in a particular part....and to troubleshoot inevitable misinterpretations around arrangements (PO-09)

Following the cluster activation, mobilisation and synthesis was also undertaken by the external broker, according to one FGD:

… [cluster facilitator] used to send me five to six mails every day.... I would say ‘please do not send so many mails as I do not read so many mails’.... but the commitment of sharing and investment from [cluster facilitator] was there and it was great”

Other FGD participants also named regular communication from the broker as an important factor to mobilise the cluster around particular issues.

#### Charismatic nodes and legitimate nodes

Charismatic nodes from within the cluster were important in recruiting to the cluster and generating enthusiasm for the model. These nodes, often referred to as stars, have high degree centrality or multiple linkages to other nodes (HCHDP in Figure 1).

Charismatic nodes may have little direct authority over network members except that derived from their referential status. French terms this *referential power* where influence is derived from respect or credibility [24]. One FGD dialogue demonstrated the importance of charismatic figures in the cluster’s activation:

…it is from their heart that they thought of CHGN-UKC and I know Dr L[de-identified] and I know Dr D [de-identified] and we have a very high regard for them (FGD-A2a). Yes these people have already worked here and we know them and their work also. So everyone wants to meet them and talk to them (FGD-A2b). I know the first community project is Share which was started by L[de-identified]..... so we have very high regard for him. He had started all these projects and most of the Christian projects in Dehradun (FGD-A2b)

Dr L was experienced in community health in India and his reputation acted as a hook for many organisations. Other cluster members were also identified as having magnetism, facilitating cluster activation and mobilisation.

In activating the cluster, the Participant Observation notes demonstrate how the facilitator sought the support of legitimate nodes, that is, stakeholders with positions in the community such as key religious leaders or those with government appointments. These legitimate nodes were integrated into the core of the cluster by allowing them responsibility for an aspect of the network meeting and activity. In particular, it was their engagement of senior (legitimate) staff from cluster programs that allowed growth and mobilisation.

We do have formidable stalwarts who are the cluster's driving force: they have power in the community of community health and primary healthcare. They are on various committees and some of the smaller programs have to answer to them sometimes (PO-09)

The legitimacy from the attendance of program leaders was crucial in making substantive commitments to intentional networking.

#### Bridging nodes

Nodal bridges were found to be particularly important in the cluster development. A bridge exists where different segments or cliques within a network are linked together by one common node or bridge. MA and M, for example, link together two cliques that would not otherwise have *activated* into the cluster (See Figure 1).

Such bridge spanners facilitated the natural growth of the cluster. For example, the FGD revealed that one node had joint appointments on Viva health network and Cana HIV network. The Participant Observation notes described how such bridges facilitated the activation of unique players into the network and provided links to help realise the cluster objectives.

He has facilitated us to link with five new programs which have helped us [the cluster] in our work. It is not just about number but linking in these new players helps us in our objectives.

### Ties and homophily

This is the links between different nodes. It represents the “glue” that brings the nodes together into a network. Homophily, the tendency to associate and bond with similar others, has been highlighted in an array of network studies and was an important explanatory factor for the development of the Uttarakhand Cluster.

#### Ideology/faith/values

In each of the FGDs common faith was identified as the pre-eminent tie in the activation and synthesis of the cluster. Common faith was also viewed as a motivator for sharing:

We formed CHGN-UKC as a like minded people, we know each other well and we know our philosophy so we all come from the same background and that is why it was easy for us to form this cluster (FGD-A2a)…and that [cluster] develops only when people have faith and they love GOD… they have a fear of GOD. In a secular cluster nobody shares because they have competition among them and also so much jealousy so we fight etc. (FGD-A2b)

Another participant shared how the faith element had helped resolve conflict and bring them back into communion:

If I had some grudges against that person, these things are cancelled if I sincerely commit myself to prayer. Through the cluster meeting I got an opportunity to build my relationship much better with many other people with whom I probably was having a bitter relationship and now that turned into a better relationship so it is an achievement because of CHGN-UKC (FGD-A2).

One cluster program manager whose program only nominally identified with the common faith nevertheless professed that a commonality of faith was the crucial factor for their involvement in the cluster. The value base, he explained, meant that he felt safe.

#### Common purpose

A recurrent theme that programs espoused was cooperation around a common vision. The cluster referred to a shared mission of bringing healthcare and services to those people most in need in developing countries:

The idea [behind the cluster] is transformation and my involvement in the cluster is to strengthen the transformation dynamic taking place in the community and in individual lives. I know that it [cluster] is really changing the lives of the people and it is helping them to get out of their problems (FGD-C2).

Other participants supported this observation. Working together, they felt, enabled them to meet common strategic objectives, and three participants cited the cooperation around the common objective of tobacco control.

The common philosophy shared by cluster members was to utilise a community level approach and this was reflected in the background vision document which asserted to “Believe in the value of community health practices, and the training/empowerment of Community Health Workers”. This discourse was observed to unite them, especially given that they exist in a health system which traditionally favours hospital based health care over community health.

#### Geographical homophily

It was evident that being geographically close to Dehradun was important for potential members to *activate* into the network. One NGO who was over four hours from where the cluster was based commented:

I consider myself too far [to contribute to the cluster] (FGD-A1).

A common geographical locality has helped mobilise participants into the cluster. It was clear that the programs isolated from the state capital city were difficult to activate and synthesise into the cluster.

#### Social and familial homophily

A recurrent theme was the importance of pre-existing relationships in activating and synthesising organisations into the cluster. The FGD participants explained how friendships with staff from other programs encouraged them to join and continue participating:

We are very happy that after a long time we all could come together. We used to meet together and now it has started again so we are very happy and we have time to talk together and share (FGD-A1).

A number of examples were described where relationships with staff from different NGOs began when they had previously worked on the same program. One key informant had worked with two NGOs before starting his own NGO, and now all three NGOs are cluster members.

Whilst a number of staff were related, others were in effect related in that they had been raised together in the same charitable home for disadvantaged children. Such social and familial homophily promoted trust amongst various cluster members as described in an FGD:

…We are not suspicious about them because we know them. We know their commitment.... but if any new outsider comes to us then we will be suspicious, but we know these people so we are not suspicious about them (FGD-A2)

### Ties and self interest

The objectives of the cluster aimed to benefit individual programs. For example:

To improve the managerial and technical capacity of individual programs, and so improve the ability of the cluster programs to apply for grants and improve the quality of their programs.

The potential for networks to provide a framework for facilitating block funding was clearly a motivating factor for potential cluster participants:

Though they [small programs] have no financial problems they still do not have enough people with expertise to apply for large grants. So as a cluster once we are a registered society we can collectively apply for funding (FGD-A2a). It is happening all over the world as these days one single organisation cannot get funds. Take the example of Global Fund. Many organisations have to join together and one single organisation cannot take fund....that is why it is good that cluster helps small organisations to come out (FGD-A2b).

Participant observation and other correspondence also revealed future opportunities as important in motivating initial involvement. Email correspondence with one informant claimed:

One very important thing is incentives or financial gain. We were talking about people coming together to receive and share ideas.... but basically it’s all for financial gain (FGD-A1).

In addition to fundraising, a number of cluster members identified opportunities for resource generation, training and capacity building as reasons for joining the cluster:

Incentives to club together? We are not talking only in terms of money but it is also building up people and providing them a chance to grow.... so it is not only monetary benefit but it is also building people (FGD-A2).

In the FGD, various programs, particularly smaller ones, expressed a desire to learn from other cluster programs:

Also I hope to get the experience of this group because all of them are so rich in their experience in the field of community health and they are working in this field more than thirty years (FGD-A2a). Yes that’s right, the only interest is learning from one another and we are getting help from one another so that is the strength of this network (FGD-A2b).

Finally, there was a belief that working together would allow individual programs greater access to resources:

And there is a benefit of sharing as we come to know who has what and can draw more benefit (FGD-C1a) …from sharing round our resources (FGD-C1b).

These findings reflect a belief amongst the cluster NGOs that they would individually benefit in terms of practical resources, trainings and tools the cluster would generate.

Interestingly, it seems that although self interest was an important factor for joining the cluster, in the second year of focus groups and Participant Observation, self interest was less predominant. Friendship, charismatic leaders, and a common purpose remained significant themes.

### Effectiveness

The cluster objectives, in summary, are to cluster together to address program gaps, increase program visibility and effectiveness, and maximise engagement with the formal sector. FGD participants believed that in the first year:

…we have done pretty well and we are doing everything we said we would do [in the action plan]

A recent article in the CHGN newsletter outlines the progress of the Uttarakhand Cluster:

…Funding to research the [cluster] model has been received from the Australia-India Council and NHMRC. They [the cluster] have recently won a grant from Mazars Trust Fund to develop and formalise their training program. They will be undertaking trainings in tobacco control, disability awareness, IT training, and in the SALT [Sensitisation, Appreciation, Learning, Transfer] methodology.

#### Increased program capacity

A number of the programs reported an increased program capacity since the cluster formation:

For us it was a new opportunity as until now .... we did not know… who was around us. Now we have a confidence and the number of our volunteers has grown. We were working with three volunteers and now we have fifteen people who can club in with us. So this is a very good benefit by getting connected with the cluster (FGD-C2).

These are early signs that networking is increasing the workforce on which projects can draw. An area where nearly all cluster NGOs have been able to grow their capacity through the cluster is in tobacco control:

We showed the program in the villages. Yes it was very, very helpful and especially in the schools as we could tell the students. In the schools we do not have to organise them as they are already organised. We just need to teach them and they grasp the message very nicely and also carry the message to their home so it is a wonderful idea (FGD-A2).

#### Improved connections

There was evidence of increased connections, although this was also an area identified for further improvement. Prior to the cluster formation, many programs had not met despite undertaking similar activities in the same region, but the cluster has changed this:

It is only through the cluster that we have come to know each other. People who were never meeting for 15 years are now meeting…this network is the platform (FGD-C2b). By the cluster, people have come to know one another, and who is doing what, so it is good that in the cluster we have developed the relationships. And there used to be a gap among us but now that gap is finished, now everyone talks to everyone and shakes hands so we know who is this brother and who is this sister (FGD-C2c).

Improved linkages to academic institutions like the University of Melbourne were also noted. Observations from the cluster meeting also revealed that to facilitate communication and linking the cluster had established:

· Email lists (chgn-ukc-consultants@googlegroups.com and chgn-ukc-Cluster@googlegroups.com)

· Website (http://www.chgnukc.org)

· Cluster newsletter

#### Collaboration and resources sharing

Beyond just increased interaction and communication there was early evidence of active collaboration:

Yes we all are under one banner and before we all were running in different directions but now we all have one destination. We have systematic approaches and common objectives and we have learnt and now we are cooperating (FGD-C1).

From the textual data explored, a number of other collaborative activities had occurred. Two examples of a bilateral collaboration initiated through cluster meetings were noted. In one case two cluster members met at a cluster meeting and developed collaborative links:

We are asking him to use his agricultural skills to do a kitchen garden for us. We will pay for the land and give him free rent in return. We have then asked him to host our staff to the garden so he can teach them. This way we gain....we learn from when he teaches us…and he gains as he farms it (PO:10).

Despite these areas of active collaboration the FGD group concluded that:

We should have had more exposure to each other’s programs and what we have done is good but much more of the same could have been done.

Resource sharing amongst programs was perhaps the most referred to cluster networking activity in the FGDs. A resource sharing meeting was arranged and ideas and resources were shared about goat keeping, disability trainings, applications for HIV programs and agricultural programs. One cluster member noted that:

Growing as a network is great because in the last two or three meetings we shared resources and other things. I found it very useful because I have been before in many networks and I am presently in many and I do not see them sharing with one another.... but this cluster has really given us an opportunity to share, exchange and interact so it was a very good meeting (FGD-A2)

At one cluster meeting, C. Hospital, H. Hospital and O discovered they were all developing training programs for the Village Health and Sanitation Committees. Subsequently they shared training materials:

“This month onwards we will be doing trainings for … the Village Health and Sanitation Committees. For this training we have some resources from H [identity withheld] also, because they are doing these trainings on a regular basis. We are in touch with O [identity withheld] also. Hope the sharing of resources will strengthen the cluster (PO-10).

#### Generating novel programs and solutions to ‘wicked’ problems

Networks, according to Agranoff, a social network theorist, help bridge organisational information gaps and asymmetries [8]. A number of instances were recounted where cluster members helped other cluster members overcome difficult problems and explore new ideas. H program helped C with a HIV application, O helped M explore new areas to work. When the programs explored new ideas together the potential was significant as the facilitator described:

The beauty about the cluster is the amazing potential of the unknown chemical reaction. The cluster is like a unique mixture of different chemicals, with an added catalyst. You will likely get a reaction…but we do not know what it will look like....The outcome objectives need to be broad to allow space for the “reaction” to expand into a shape of its own (PO-10).

One such area is in tobacco control, an area in which none of the programs had previously worked, but with the outside catalyst and corporate capacity, the cluster projects have been able to begin new programs in tobacco control. The cluster members expressed that they have been able to approach problems together:

All of us are different and we all have different thinking about the methods of the work, and when we come together ten different things come together and when ten people think over those ten different things we get the solution for the problems…and we all learn by it a lot (FGD-C2).

This contrasts with the previous situation where many programs felt alone and inadequately skilled in tackling new issues.

#### Visibility and advocacy

There is evidence that the cluster network has emboldened members to advocate on issues and given them a platform to be heard by non-cluster NGOs and government programs. Cluster members have utilised the platform to advocate on TB, tobacco control and disability.

Although we do everything very honestly it is very difficult alone in Tehri Garhwal… but as a cluster we have strength: a strength of maybe twenty or ten organisations. So we can work and fight for health (FGD-A2).

The cluster collaboratively produced and publically launched an anti-tobacco DVD which was considered to not only increase the visibility of member programs, but also give them confidence to engage with other key health players in India:

After the launch the government now knows more about CHGN-UKC …well at least the health department knows us (FGD-A2).

Now we have such a confidence that we can even work with government. Previously we were not able to deal with the government but now the government has started to give us small projects. Now we are able to do the works which are instructed by the government (FGD-C2).

At the DVD launch the director of national programs in the government proffered an open invitation to the members to visit him, and a number of programs did. The cluster coordinator reportedly told him:

I said ‘If any program comes to Uttarakhand in which we, the CHGN-UKC, can be instrumental please let us know. You can use this resource.’ He said ‘yes’ but still I do not know.....The cluster capacity helps me to go to government officers and explain what we are doing (FGD-A2).

### Barriers to networking

There were a number of resource constraints identified (staff, time and money), and attitudinal barriers (mistrust, uneven contributions, zero-sum mentality) to the activation, framing, mobilisation and synthesizing of the Uttarakhand Cluster.

#### Staffing

It has been difficult for the cluster to engage with dedicated and qualified staff members from the cluster programs:

Sometimes programs do not have such staff and at other times they are preoccupied with survival activities. If the member organisations are weak from within, networking and learning among cluster members will also be weak (PO-09).

Compounding a lack of qualified staff to drive the cluster was departure of staff who were strong advocates for the cluster. Participant observation revealed that a previous director had been supportive of the cluster, but the incumbent director did not comprehend the vision behind the cluster. This is particularly damaging if the departing staffer was the broker or bridge between parts of the network.

#### Money

Financial viability was a problem for a number of the cluster members and limited their involvement and investment in the cluster programs. One participant observer noted:

It is evident as one speaks to most participants, there is a consensus on the vulnerability of their very existence: their ability to engage is linked so closely to availability of funds and successful running of programs (PO-09).

The unavailability of funds discouraged NGO involvement in the cluster due to the costs of attending meetings, the membership fees and the small transport costs. These were particularly relevant when there was no immediate tangible fiscal return from the investment:

We have limited resources and it is a problem for us as long as we do not have any returns ....but at this stage the cluster requires our contribution (FGD-C1).

Despite the indications of cluster effectiveness (above), the fiscal returns have largely resulted from informal meetings and bilateral arrangements facilitated through cluster meetings but not a direct program of the cluster.

#### Time

The most commonly identified barrier to Uttarakhand Cluster network development was poverty of time. Some participants prioritised devoting time to cluster activities:

Mr S got a global fund and he is too busy, E.H.A are just running here and there.... and Mrs S and R both have finished the ASHA training....but they are still coming for this one meeting despite the time requirement (PO-09).

However, the FGD indicated that more often cluster members tended to prioritise their own program activities. There were various comments on how little time was left after commitments to trainings, government reporting, and completing their work for donors. Given this situation, longer cluster meetings tended to have low attendance:

Visits are not done as both the parties have no time (FGD-C2a) Yes…it takes a lot time to visit and many of the organisations have not time (FGD-C2b). We can’t afford five days out for the disability training (FGD-C2a). Time is a challenge....the biggest challenge is that we are less in number but the working area is very large (FGD-C2b).

The mountainous terrain and time to travel between cluster programs was surprisingly not felt to be a barrier to networking.

#### Communication

Despite increased communication between programs (see above) communication was still a barrier to synthesis of the cluster. Inadequate usage of the intra-cluster communication channels (googlegroups, address lists, newsletters) was perceived as a barrier to cluster development:

…Another thing… we could bring two people to the DVD launch.... but we did not know about it and now it is not possible to bring any one of them (FGD-C1b). So there is a little communication gap among us (FGD-C1c).

This was despite emails and reminders going to the cluster members. However, it seems that many of the cluster programs had unreliable access to email. Furthermore, even where correspondence occurred, the concepts and details were not always grasped.

#### Zero sum game

The networking concept pre-supposes that when each program shares their part then the resources available are multiplied. A challenge given by a meeting participant summarises this approach:

We are unique in our desire to serve each other in love, giving of our gifts for others. It will be a feast if there is a focus on giving. Can we as individual programs focus on giving before getting instead of getting before giving? Promoting this attitude of giving is the greatest challenge. If each cluster member focuses on giving then cluster members will be blessed....ten-fold. However, if we come with an attitude of “what can I get” then the cluster is lost! Unfortunately the missionary history of many of these programs has promoted a history of “receiving” (PO-10).

However, according to the FGD, despite increased cooperation, zero sum thinking still limits the effectiveness of the cluster. That is, if I share my knowledge then I risk losing my competitive advantage:

Many programs come from a background where secrecy is a survival technique. This is a zero sum game attitude: “If we share information they will take our support and work” (PO-E10).

This led to disproportionate participation in the cluster which resulted in some ill-feeling from those who contributed more:

At the meeting the other day, many organisations did not come, so other organisations might say that why should we come? Our time is being wasted. There are some people who come from very far off places, spending the entire Sunday travelling, so this can be a problem (FGD-C1).

Whilst informal sharing of ideas is relatively risk free, more formal collaboration is accompanied by risk. Ultimately their vulnerable state added to their caution to share the little resources that they had:

The fragile state of the participants makes one concerned about their willingness to actively participate in an unfamiliar or even experimental approach of the cluster (POE-09).

#### Mistrust

As illustrated above, there were indications from Participant Observation that the cluster framework had facilitated cooperation between programs which were previously suspicious of each other. As one FGD participant commented, the:

"“Bitter relationship [has].... turned into a better relationship”."

However, some cluster NGOs remained reticent about close collaboration. In the FGDs, concern was raised that through intra-cluster cooperation, or extra-cluster cooperation with the government, they might become embroiled in corruption, either actual or alleged:

How can we trust the government or even another secular program? (FGD-C1a). We have had bad experiences and we don’t want to risk our reputation with them (FGD-C1b).

Some programs with conservative religious beliefs were hesitant to engage with government institutions - who were perceived to be “hostile towards FBOs”- whilst programs with more liberal beliefs favoured engagement. These ideological differences generated some mistrust between programs.

## Discussion

This case study provides useful insights into the facilitators of, and barriers to, network formation, and indicates the functions that a network can fulfil. Multifarious factors were involved in activating, framing, mobilising and synthesizing the cluster. These included key brokers and nodes (network players) with a high degree of network centrality and nodes who bridged between different organisational groups. Ties (relationships) that were found to promote clustering included homophily around common faith, common friendships, geographical location and common mission. Self interest drives many of the programs to be involved and so cluster effectiveness is important. The research indicates that in less than two years, the cluster has effectively improved access to funds and trainings, promoted visibility and credibility, and increased the program capacity. The barriers can be summarised as relating to risk aversion, including the risk of cooperating and investing, and inadequate resources to permit investment in the cluster.

### Facilitating factors

The importance of brokers in providing an environment for favourable and productive interaction is a finding supported by Agranoff’s work, where he details the importance in network formation of creating an environment for favourable and productive interaction [16]. In another book he explains that in network formation “structure follows strategy” [8]. This study supports this finding: the success of the Uttarakhand Cluster was not a random association but underpinned by strategic brokerage. The analysis and weight of responses suggests brokerage was one of the most significant factors facilitating the network formation.

The brokers actively sought to integrate legitimate, charismatic nodes to help create this favourable environment for cluster mobilisation, framing and synthesis. These nodes were determined to be of relative high importance in drawing in additional programs to the cluster. This effect may well be of greater importance in India where organisational culture tends to prioritise hierarchy [25].

Bridging nodes were also important in drawing in different cliques: that is, other groups of programs. However, when a network relies on individual bridges but has little redundancy (alternative ties between groups), structural holes result as the network becomes over dependent on a few key bridging nodes [5]. For example, the bridge spanning CHGNUKC (coordinator), H and MA had little redundancy and if they were removed from the network the link to an entire clique might be lost.

Homophily was demonstrated to be important in drawing programs into the cluster, but it is difficult to distinguish the effect of each type of homophily (faith, geographical, familial, social) due to the overlap. For example, a family may have the same faith and be socially linked also. There was also a tendency to overstate faith commitments to project a pious image. Homophily was particularly important in India, a trust based society, as the people you can more likely trust are those with whom one is already in relationship.

Common goals were clearly motivating for cooperation but these were constantly moving. For example, in the mobilisation phases, the direction of the cluster shifted towards development in a broader sense, even incorporating NGOs with school and education foci. At times the common goal was actually self interest which is potentially powerful when the self interest of multiple programs results in the same action/s. Given that self-interest was a motivating factor, the benefit needs to be continually reinforced and demonstrated.

### Barriers to networking

Building a network of sharing is counter-cultural to many of these programs which have previously existed in organisational silos. The novel idea of sharing resources runs counter to the accepted wisdom of zero sum game where projects are hesitant to share resources, believing that sharing represents a loss for the program sharing the resource. This has led to reluctance to freely share and therefore unequal participation.

Many of the barriers to effective networking were related to risk aversion from already overstretched NGOs unwilling to risk allocating resources, staff and time to the non-core cluster. Although sharing resources and collaborating may be rewarding, there is a risk to the program in trusting other similar programs which may have a different modus operandi and interpretation of faith. This explains the reticence to collaborate closely with programs they did not know very well. Coleman explains that trust is a function of dense and closed networks where the players are well known, whereas the cluster represents a looser association. Additionally, in what is perceived as non trust-based Indian society [5], loose networks and collaboration exposes members to real risks.

Interestingly, the problems that the cluster hopes to address are the very same problems limiting program involvement in the cluster. Members had inadequate resources (money/time/staff) for substantive involvement in the cluster even though such involvement might be an avenue for training more staff and engaging with donors. Instead, programs tended to maximise short term gains at the expense of capacity building activities such as networking. When busy, financially stressed or understaffed, which was nearly all the time, survival functions were prioritised over cluster activities.

Given the risk averse nature of programs in this area, delivering initial success or ‘quick wins’ through the cluster was thought to be essential (FGD-C1). In light of this, the cluster determined to link together to generate a resource (anti-tobacco DVD) and then launch it at a high level event. This “getting our hands dirty, together” (PO-09) achieved a tangible outcome which they could not have achieved independently. However, there was much room for more substantive sharing or pooling of resources.

### Study limitations

This analysis is an early indication of the effectiveness, or otherwise, of the cluster model. Follow-up surveys will provide better evidence for the impact of the cluster model on health outcomes.

Although the general principles can be applied to other network settings, generalisability will be limited by various contextual factors. This study is also limited in disentangling the effect, and therefore the importance, of any one individual factor promoting networking. The literature suggests that network formation is a multi-stranded process and that factors important in the activation of a network may be quite different to the various factors leading to network mobilising and synthesis [7].

The study results are limited by the presence of social acceptability bias in that members were unlikely to admit to conflict or selfish motivations for their involvement in the cluster. For example, the importance of self interest (money, resources etc.) was felt to be understated due to social acceptability bias: stating self interest as a reason for being involved would be viewed as unacceptable by cluster members.

## Conclusion

When activating, framing, mobilising and synthesizing such clusters, brokerage is important and can help massage the process and identify key nodes who are in legitimate positions, have a high degree of centrality in the field, and possess charismatic appeal.

Capitalising on *ties* is important in uniting a network like the cluster. It would seem important to identify and exploit homophily around common faith, common friendships and common geographical location to knit networks together. Likewise a common vision brings programs together and operationalising this vision by collaborating on a common activity can provide quick wins and strengthen the network.

The broker of a network of community health programs might need to be cogniscent of the members’ limited resources (time, money and staff) and potential risk aversion in investing these resources. Building a network of sharing may be counter-cultural to many small community programs which have previously existed in organisational silos, and trust needs to be promoted. Ultimately programs in a network, however magnanimous their motivation, are driven by an element of self interest. To grow networks it seems evident that the effectiveness of such a network needs to be demonstrated and continually reinforced. The effectiveness of the cluster had been demonstrated already in access to funds, visibility and credibility, and capacity building and training.

These results are informative for networking development in situations where groups of NGOs work alongside each other in similar health and development programs, especially where they have common values. This data has already been instructive for CHGN as they facilitate clusters in Kenya and Bangladesh and consider establishing clusters in Burma, Sierra Leone, Ethiopia and North Malawi.

## Competing interests

The authors declare that they have no competing interests.

## Authors’ contributions

NG conceived of the study, and participated in its design and coordination. NG oversaw the implementation of the study on the ground and undertook the thematic analysis. PR developed the study protocol and reviewed various iterations in response to the ethics committee process. PR provided oversight to the entire project and provided feedback at each point as the data was collected and analysed. MP participated in the initial design and was responsible for undertaking the interviews and FGDs. He coordinated the collection of the data on the ground. All authors reviewed the numerous drafts of this article and have read and approved the final manuscript.

## Pre-publication history

The pre-publication history for this paper can be accessed here:

http://www.biomedcentral.com/1472-6963/12/206/prepub
